# Body fat percentage is independently and inversely associated with serum antibody responses to SARS-CoV-2 mRNA vaccines

**DOI:** 10.1038/s41598-022-21884-z

**Published:** 2022-11-10

**Authors:** Jeremy B. Ducharme, Zachary J. McKenna, Zachary J. Fennel, Roberto C. Nava, Christine M. Mermier, Michael R. Deyhle

**Affiliations:** 1 Department of Health, Exercise and Sports Sciences, University of New Mexico, Albuquerque, NM USA; 2 Research Division, Joslin Diabetes Center, Boston, MA USA; 3 Harvard Medical School, Harvard University, Boston, MA USA; 4 Institute for Exercise and Environmental Medicine, University of Texas Southwestern Medical Center, Dallas, TX USA; 5 Department of Cell Biology and Physiology, School of Medicine, University of New Mexico, Albuquerque, NM USA

**Keywords:** Diseases, Risk factors

## Abstract

Vaccination is widely considered the most effective preventative strategy to protect against severe acute respiratory syndrome coronavirus-2 (SARS-CoV-2) infection. An individual’s exercise habits, and physical fitness have been shown to impact the immune response following vaccination using traditional vaccine platforms, but their effects are not well characterized following administration of newer vaccination technology (mRNA vaccines). We investigated these effects on the magnitude of antibody responses following SARS-CoV-2 mRNA vaccination while accounting for known covariates (age, sex, time since vaccination, and the type of vaccine administered). Adults of varying fitness levels (18–65 years; *N* = 50) who had received either the Moderna or Pfizer SARS-CoV-2 mRNA vaccine between 2 weeks and 6 months prior, completed health history and physical activity questionnaires, had their blood drawn, body composition, cardiorespiratory fitness, and strength assessed. Multiple linear regressions assessed the effect of percent body fat, hand grip strength, cardiorespiratory fitness, and physical activity levels on the magnitude of receptor binding domain protein (RBD) and spike protein subunit 1 (S1) and 2 (S2) while accounting for known covariates. Body fat percentage was inversely associated with the magnitude of S1 (*p* = 0.006, *β* = − 366.56), RBD (*p* = 0.003, β = − 249.30), and S2 (*p* = 0.106, *β* = − 190.08) antibodies present in the serum following SARS-CoV-2 mRNA vaccination. Given the increasing number of infections, variants, and the known waning effects of vaccination, future mRNA vaccinations such as boosters are encouraged to sustain immunity; reducing excess body fat may improve the efficacy of these vaccinations.

## Introduction

Vaccination is widely considered the most effective preventative strategy to protect against severe acute respiratory syndrome coronavirus-2 (SARS-CoV-2) infection. While traditional vaccine platforms include inactivated or attenuated virus, or recombinant viral proteins, two prominent vaccines to combat SARS-CoV-2 infection, Pfizer (BNT162b2/Comirnaty) and Moderna (mRNA-1273), are both lipid-encapsulated mRNA-based vaccines. This newer vaccination technology has the notable advantage of eliciting higher antibody responses while also being more rapid to produce in large quantities compared to more traditional methods^1^.

Although the Moderna and Pfizer SARS-CoV-2 vaccines appear to be highly effective at preventing COVID-19^1^, some individuals who have received the vaccine still contract the disease. For example, of 4,468 patients infected with SARS-CoV-2 in the Houston Methodist health care system between late November 2021 through January 5, 2022, 2,497 (55.9%) had been fully vaccinated (e.g., > 14 days after receiving two doses of the Pfizer [73%] or Moderna [22%] mRNA vaccines or one dose of the J&J [JNJ-78436735] recombinant adenovirus vaccine [5%])^2^. Similarly, previous vaccination attempts for other viral diseases, such as influenza, have also observed breakthrough illnesses in individuals who have been vaccinated^3^. The evolution of SARS-CoV-2 resulting in the advent of multiple variants has contributed to continued SARS-CoV-2 infection, even among vaccinated and previously infected individuals^1,4^. To combat these variants, it is crucial to understand the wide inter-individual variability that alters the magnitude of protective immunity acquired after vaccination. The individual response to vaccinations varies substantially depending on the type of vaccine administered, age and sex of the individual, and time since vaccination^1,5^. For example, recent studies have demonstrated that protective immunity can wane within 6 months post vaccination increasing the risk of SARS-CoV-2 infection^6^. Importantly, modifiable factors related to one’s lifestyle such as an individual’s exercise habits, and physical fitness have also been shown to impact the immune response following vaccination. Researchers have demonstrated with previous vaccination programs that individuals who exercise regularly^7,8^, as well as those that performed an acute bout of exercise within 30 min of vaccination^9^, have improved antibody responses compared to their age-matched controls. Moreover, obesity is associated with impaired immune responses following the influenza vaccine^10–12^ and COVID-19 vaccines^13^, whereas weight loss is associated with improved responses^14^. Together these findings demonstrate that levels of physical activity and body fat can affect the efficacy of vaccination.

General lifestyle recommendations including regular exercise to support immune function amid the COVID-19 pandemic have been issued^15^, but the role of lifestyle factors on the prophylactic immune response to SARS-CoV-2 vaccination remains understudied^16^. A further element of uncertainty is that two prominent SARS-CoV-2 vaccines currently being distributed in the United States and other countries are mRNA-based vaccines^17^. Previous researchers have demonstrated that physical activity and obesity can contribute to heterogeneity of the immune response following vaccination with vaccines involving inactivated, attenuated, or recombinant viral protein vaccine platforms. Although a recent study showed that an acute bout of exercise after mRNA vaccination boosts antibody responses^9^, the effect of regular physical activity and body fatness on the antibody response following mRNA vaccination is not known. Additionally, the impact that other components of fitness such as muscular strength and cardiorespiratory (aerobic) fitness may have on prophylactic immune responses to mRNA SARS-CoV-2 vaccines are also unknown. Therefore, the purpose of this study was to address this gap in the literature by investigating the effects of exercise habits and several aspects of physical fitness on the magnitude of the antibody responses following SARS-CoV-2 mRNA vaccination. Given that previous researchers have shown that age, sex, time since vaccination, and the type of vaccine affect the antibody response to vaccinations^1,5^, the current study aimed to evaluate the effect of exercise habits and components of physical fitness on the antibody response following vaccination at the individual level while accounting for these covariates.

## Materials and methods

### Experimental design

Using a cross-sectional study design, participants who had received their second dose of either the Moderna or Pfizer SARS-CoV-2 mRNA vaccine between 2 weeks and 6 months prior, arrived at the Exercise Physiology Laboratory for a single day of data collection in which they completed health history and physical activity questionnaires, and had their blood drawn before having their body composition, cardiorespiratory fitness, and strength assessed. All data collection was completed between April 2021 and September 2021 meaning that boosters for the Moderna and Pfizer SARS-CoV-2 mRNA vaccines were not yet available for our participants.

### Participants

Data were collected on 60 participants (men, *n* = 26; women, *n* = 34) of varying fitness levels including university students, faculty, staff, as well as local community members. Participant demographics are presented in Table 1. Adults between the ages of 18 and 65 who received their first and second dose of either the Moderna or Pfizer SARS-CoV-2 mRNA vaccine between 2 weeks and 6 months prior to visiting the Exercise Physiology Laboratory for data collection were eligible for the study. Participants were ineligible if they did not meet the inclusion criteria or were (a) experiencing symptoms of COVID-19 (cough, fever, shortness of breath, loss of taste or smell, chills, body aches, sore throat, diarrhea), (b) had a diagnosis of an immune-compromising condition, or (c) taking a medication that alters heart rate. Participants were apparently healthy and had no known diseases or symptoms. All research procedures were approved by the University of New Mexico Institutional Review Board prior to starting this research (IRB # 05321, approved on April 17th, 2021). This research was performed in accordance with the Declaration of Helsinki. Each participant provided written informed consent before beginning the study.

**Table 1:** Participant demographics.

	Men (*n* = 24)	Women (*n* = 26)	Total (*N* = 50)
Age (years)	36.8 ± 13.2	41.7 ± 15.1	39.3 ± 14.4
Height (cm)	179.3 ± 6.9	164.1 ± 6.5*	171.4 ± 10.1
Body mass (kg)	80.3 ± 11.3	62.4 ± 9.7*	71.0 ± 13.8
Body fat (%)	16.12 ± 7.1	25.4 ± 5.7*	20.9 ± 7.9
Estimated VO_2max_ (ml/kg/min)	50.0 ± 7.1	37.5 ± 7.4*	43.5 ± 9.6
Hand grip strength (kg)	52.4 ± 12.1	31.8 ± 5.2*	41.7 ± 13.8
Relative hand grip strength	0.66 ± .16	0.52 ± 0.10*	0.59 ± 0.15
MET (min/week)	4591.5 ± 5531.4	4758.7 ± 5699.1	4678.4 ± 5619.1
Time since vaccination (days)	113 ± 60	118 ± 67	116 ± 64

*MET* Metabolic equivalent of task.

Estimated $\dot{\mathrm{V}}{\mathrm{O}}_{2\mathrm{max}}$=maximal cardiorespiratory fitness estimated using a submaximal test. *Denotes significantly different than men *p* < 0.05.

### Physical activity questionnaire

The short International Physical Activity Questionnaire (IPAQ) was used to gather information about the participant’s exercise and physical activity habits. The short IPAQ is a widely used questionnaire that provides a reliable and valid assessment of physical activity habits^18,19^. The questionnaire consists of seven questions related to the amount of time an individual spends exercising or working at low, moderate and vigorous levels of exertion over a typical 7-day period. When completing the questionnaire, participants were asked to recall their average 7-day level of physical activity that they had engaged in over the previous 6 months. The data gained from the questionnaire was processed accordingly to determine the average self-reported metabolic equivalent of task (MET) minutes/week^18^.

### Blood sample collection

Blood samples were collected through venipuncture of an arm vein into serum separator Vacutainers®. Blood samples were centrifuged at 1600xg for 15 min in 4 °C to separate serum. Samples were stored in 1 mL aliquots in a − 80 °C freezer until later analysis. Serum samples were assayed for antibodies that react with the following four SARS-CoV-2 proteins: receptor binding domain protein (RBD), spike protein subunit 1 (S1), spike protein subunit 2 (S2), and nucleocapsid (N) via MAGPIX multiplexing (Luminex xMAP Technology, San Diego, CA) and were reported according to their median fluorescence intensity (MFI). Because the SARS-CoV-2 vaccines used in this study only deliver messenger RNA that encodes for the spike protein, participants whose samples were positive for N protein-reactive antibodies were deemed to have been previously exposed to the live SARS-CoV-2 virus and were removed from further analyses. Intraassay coefficient of variations for S1, S2, RBD, and N antibodies were 5.35%, 4.53%, 8.95%, and 11.85%, respectively.

### Anthropometric measurements

Participant height (cm) was measured using a stadiometer (Holtain Limited, Crymych, Dyfed, Great Britain) and body mass (kg) was recorded using a digital weight scale (MedWeight MS-3900, Itin Scale Company, Brooklyn, NY, United States). Participants’ body density was estimated using 3-site skinfold measurements and equations for men (chest, abdomen, and thigh^20^) and women (triceps, suprailiac, and thigh^21^). These measurements were used to estimate participants’ percent body fat (%BF) using the Siri^22^ equation.

## Cardiorespiratory fitness

Maximal cardiorespiratory fitness ($\dot{\mathrm{V}}{\mathrm{O}}_{2\mathrm{max}}$) was estimated via the Ebbeling et al.^23^ sub-maximal walking test on a motorized treadmill (Precor® C966, Woodinville, WA). Participants were fitted with a wireless heart rate monitor (Polar, Oulu, Finland) with the transmitter placed around their chest. Following a two-minute walking warm up at a slow speed (1.5 to 2 mph), the speed of the treadmill was increased to elicit a heart rate between 50 and 70% of the participants’ estimated heart rate maximum (220-age). After the appropriate heart rate was reached, incline of the treadmill was increased to a 5% grade. Heart rate was recorded again at minutes 3 and 4 during this stage. If the heart rate measurements varied by more than 5 beats per minute, the test was extended for another minute and heart rate was measured again. The final two heart rates were used to calculate estimated maximal cardiorespiratory fitness according to the Ebbeling et al.^23^ method.

### Muscular strength

Hand grip strength (HGS) of the dominant hand was measured using a manual spring-loaded dynamometer (A729-300 hydraulic hand dynamometer, Rolyan Ability One, Germantown, WI). The hand grip dynamometer was adjusted relative to the individual’s hand size, so that the bar was between the proximal and middle phalanges. While standing erect, the arm and forearm were positioned such that the shoulder was adducted and neutrally rotated, elbow flexed at 90°, and forearm in a neutral position^24^. Participants were instructed to squeeze the handle of the dynamometer with maximal effort for approximately 3 s with no extraneous body movement. This process was repeated three times with 1-min rest between trials. The highest value was recorded in kilograms and normalized to body mass (kg of grip force ÷ kg of body mass) for analyses.

### Statistical analyses

An a priori power analysis was conducted using G*Power software (version 3.1.9.6). Using a conservative estimate of effect size (*f*^*2*^ = 0.15), with one tested predictor (either %BF, relative HGS, estimated $\dot{\mathrm{V}}{\mathrm{O}}_{2\mathrm{max}}$, or MET min/week) and four covariates (age, sex, time since vaccination, and the type of mRNA vaccine received) making a total of five predictors for each model, it was estimated with an α-level of 0.05, and a power of 0.80 (1–β) that 55 participants would be required to detect a statistically significant effect of medium size on the outcome variables (S1, S2, and RBD antibodies following SARS-CoV-2 mRNA vaccination). Differences among participant demographics were evaluated using two-tailed independent sample *t* tests.

Four separate hierarchical multiple linear regression analyses were performed to assess the main effect of an individual’s %BF, relative HGS, estimated $\dot{\mathrm{V}}{\mathrm{O}}_{2\mathrm{max}}$, and self-reported MET minutes/week on the magnitude of RBD, S1, and S2 antibodies following SARS-CoV-2 mRNA vaccination. Age, sex, time since vaccination, and the type of mRNA vaccine received (Pfizer or Moderna) were included as covariates due to their known associations with prophylactic immune responses to vaccination^5,25^. These predictive models were defined as the body composition model (%BF and covariates), strength model (relative HGS and covariates), aerobic fitness model (estimated $\dot{\mathrm{V}}{\mathrm{O}}_{2\mathrm{max}}$ and covariates), and the physical activity model (MET minutes/week and covariates). Dummy codes were created for sex (0 = women, 1 = men) and type of mRNA vaccine received (0 = Moderna, 1 = Pfizer). The β coefficient for each predictor variable of interest was used to quantify the magnitude of their effect. Standard error (SE) was used as a measure of the statistical accuracy of an effect size. An α of 0.05 was used as a threshold for statistical significance. Analyses were performed using RStudio version 2022.02.2 + 485 "Prairie Trillium" for Windows. Case diagnostics were performed to ensure assumptions of linearity were met and correlations between variables were examined for multicollinearity. In all models, the standardized residuals were examined to confirm that assumptions of normality and homoscedasticity were met. Potential influential outliers in each model were examined by comparing the Cook’s distance values.

## Results

### Model building

**Figure 1:**
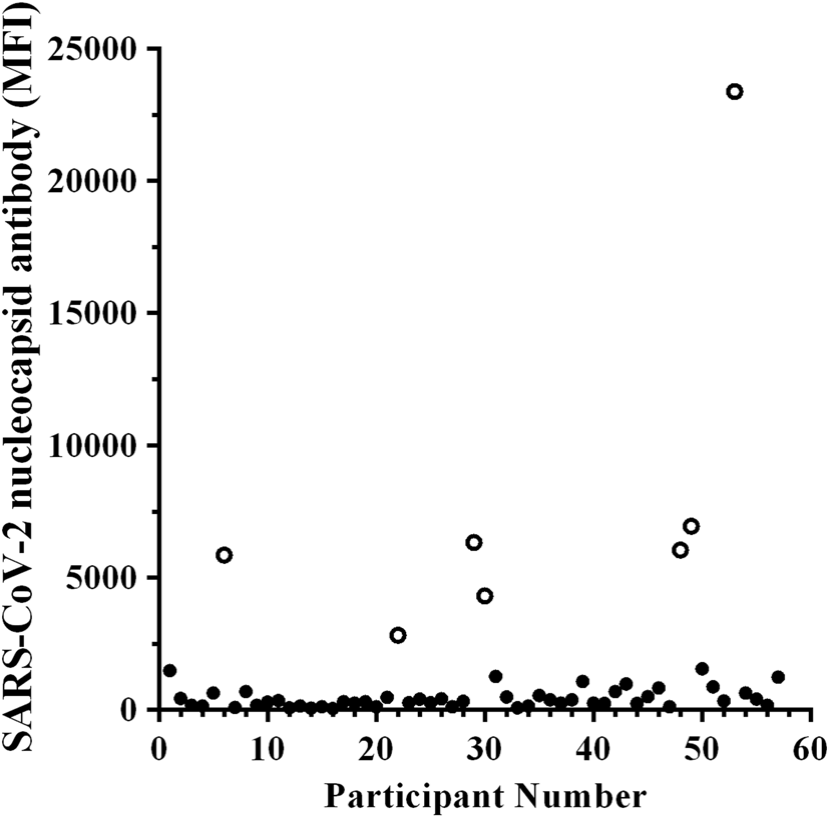
SARS-CoV-2 nucleocapsid-specific serum antibody levels amongst participants (*N* = 57). MFI = median fluorescence intensity. Open circles indicate the participants likely to have had a previous SARS-CoV-2 infection.

Case diagnostics identified two males and one female as potential influential outliers by having large Cook's distance values relative to the rest of the data set. To avoid violating the normal distribution assumption of regression analysis, these data points were excluded from further analyses. An additional seven participants were excluded from analyses as they were deemed to have likely had a recent SARS-CoV-2 infection based on high levels of antibodies against SARS-CoV-2 N (Fig. 1). Assessments of multicollinearity revealed that sex had a strong association with %BF (%BF = − 9.3 (sex) + 25.4, *R*^*2*^ = 0.340, *p* < 0.001), relative HGS (HGS = 0.14 (sex) + 0.52, *R*^*2*^ = 0.213, *p* < 0.001) and estimated $\dot{\mathrm{V}}{\mathrm{O}}_{2\mathrm{max}}$ ($\dot{\mathrm{V}}{\mathrm{O}}_{2\mathrm{max}}$=12.5 (sex) + 37.50, *R*^*2*^ = 0.421, *p* < 0.001). This meant that on average, women had 9.3% more body fat, 0.14 kg less relative HGS, and an estimated $\dot{\mathrm{V}}{\mathrm{O}}_{2\mathrm{max}}$ that was 12.5 ml/kg/min less than men (Table 1). To avoid violating the assumption of collinearity, sex was removed from the models that investigated the effect of %BF, relative HGS, and estimated $\dot{\mathrm{V}}{\mathrm{O}}_{2\mathrm{max}}$ on the S1, S2, and RBD antibody response. No association was observed between the sex of the individual and the average amount of physical activity they engaged in each week (MET min/week = − 167.2 (sex) + 4758.7, *R*^*2*^ < 0.01, *p* = 0.954). Of the participants in the current study, 70% (35/50) received the Pfizer SARS-CoV-2 mRNA vaccine while 30% (15/50) received the Moderna SARS-CoV-2 mRNA vaccine.

### Exercise habits, physical fitness, and antibody responses

**Figure 2:**
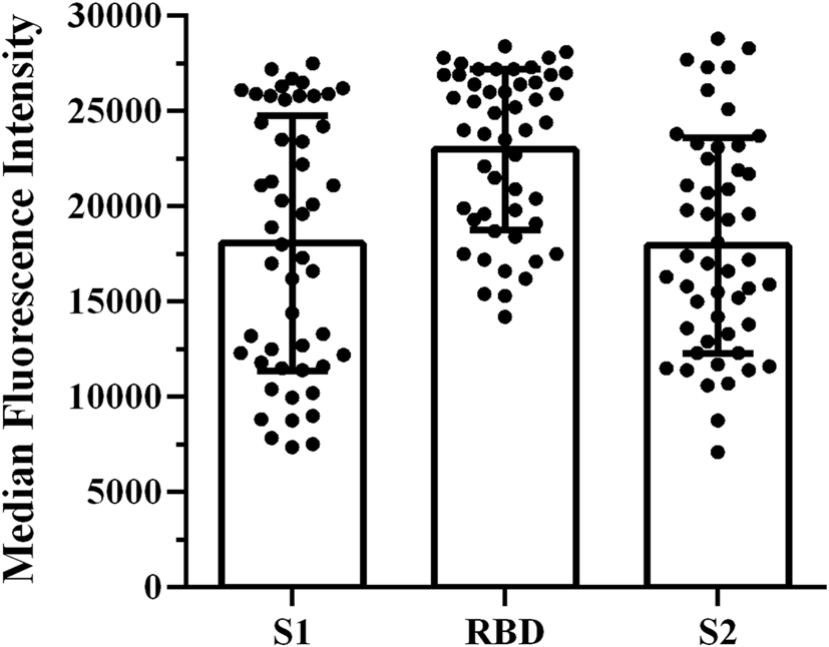
Viral protein-specific serum antibody levels in response to SARS-CoV-2 mRNA vaccination. Spike protein subunit 1 (S1), receptor binding domain protein (RBD), and spike protein subunit 2 (S2) (*N* = 50).

To illustrate the range of individual’s antibody responses to vaccination, Fig. 2 shows the magnitude of antibodies present in the serum following SARS-CoV-2 mRNA vaccination. To investigate the effect of modifiable physical fitness variables on these responses multiple models were developed.

**Figure 3:**
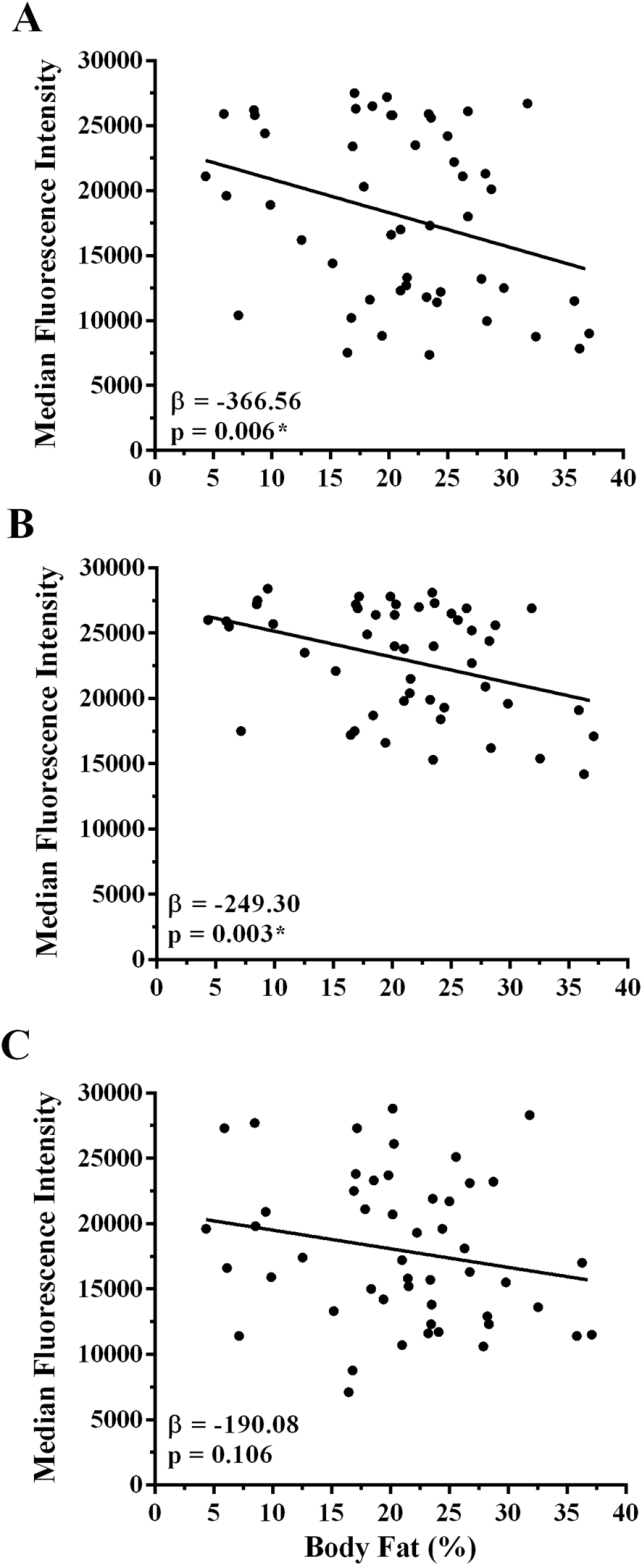
Effect of body fat on viral protein-specific serum antibody levels in response to SARS-CoV-2 mRNA vaccination after accounting for the known effects of age, time since vaccination and the type of mRNA vaccine (Pfizer or Moderna) received. Spike protein subunit 1 (**A**), receptor binding domain protein (**B**), and spike protein subunit 2 (**C**) (N = 50). *Denotes statistical significance *p* < 0.05.

The body composition model (F_4,45_ = 2.735, *p* = 0.040, *R*^*2*^ = 0.195) indicated a significant effect of %BF on the amount of S1 antibody present in the serum (*β* = − 366.56, SE = 129.2, *p* = 0.006, Fig. 3A). This meant that for every 1% increase in %BF there was a significant decrease in the amount of S1 antibody regardless of the individual’s age, time since vaccination, or the type of mRNA vaccine received. In the strength model (F_4,45_ = 0.624, *p* = 0.648, *R*^*2*^ = 0.052), no significant effect was observed for relative HGS on the amount of S1 antibody present in the serum (*β* = 1279.56, SE = 6732.23, *p* = 0.850). For the aerobic fitness model (F_4,45_ = 1.257, *p* = 0.301, *R*^*2*^ = 0.100), no significant effect was observed for estimated $\dot{\mathrm{V}}{\mathrm{O}}_{2\mathrm{max}}$ on the amount of S1 antibody present in the serum (*β* = − 199.95, SE = 128.09, *p* = 0.125). Using the physical activity model (F_4,45_ = 1.003, *p* = 0.427, *R*^*2*^ = 0.102), no significant effect was observed for physical activity on the amount of S1 antibody present in the serum (*β* = 0.27, SE = 0.17, *p* = 0.127).

In agreement with the S1 antibody response, the body composition model (F_4,45_ = 3.201, *p* = 0.021, *R*^*2*^ = 0.221) indicated a significant effect of %BF when controlling for these covariates on the amount of RBD antibody present in the serum (*β* = − 249.30, SE = 80.13, *p* = 0.003, Fig. 3B). Using the strength model (F_4,45_ = 0.681, *p* = 0.609, *R*^*2*^ = 0.057), like the S1 antibody response, no significant effect was observed for relative HGS on the amount of RBD antibody present in the serum (*β* = 1612.46, SE = 4232.33, *p* = 0.705). Similar to the S1 antibody response, no significant effect was observed in the aerobic fitness model (F_4,45_ = 0.983, *p* = 0.426, *R*^*2*^ = 0.080) for estimated $\dot{\mathrm{V}}{\mathrm{O}}_{2\mathrm{max}}$ on the amount of RBD antibody present in the serum (*β* = − 92.64, SE = 81.61, *p* = 0.262). Using the physical activity model (F_4,45_ = 1.175, *p* = 0.336, *R*^*2*^ = 0.118), like the S1 antibody response, no significant effect of physical activity was observed on the amount of RBD antibody present in the serum (*β* = 0.19, SE = 0.11, *p* = 0.081).

The body composition model (F_4,45_ = 1.299, *p* = 0.285, *R*^*2*^ = 0.103) indicated no significant effect of %BF on the amount of S2 antibody present in the serum (*β* = − 190.08, SE = 115.14, *p* = 0.106, Fig. 3C). Similar to the observation with S1 and RBD antibody levels, no significant effect was observed for relative HGS in the strength model (F_4,45_ = 0.628, *p* = 0.645, *R*^*2*^ = 0.053) on the amount of S2 antibody present in the serum (*β* = − 2342.00, SE = 5680.30, *p* = 0.682). Using the aerobic fitness model (F_4,45_ = 1.109, *p* = 0.364, *R*^*2*^ = 0.090), similar to the S1 and RBD antibody response, no significant effect was observed for estimated $\dot{\mathrm{V}}{\mathrm{O}}_{2\mathrm{max}}$ on the amount of S2 antibody present in the serum (*β* = − 153.74, SE = 108.74, *p* = 0.164). Again, like the S1 and RBD antibody response, the physical activity model (F_5,45_ = 1.144, *p* = 0.352, *R*^*2*^ = 0.115) indicated no significant effect of physical activity on the amount of S2 antibody present in the serum (*β* = 0.23, SE = 0.14, *p* = 0.120).

## Discussion

The primary aim of this study was to determine the effect of physical activity habits and multiple aspects of physical fitness—strength, aerobic fitness, and body composition—on the magnitude of antibody responses following SARS-CoV-2 mRNA vaccination. Poor responses to vaccination (e.g., lower viral protein specific antibody production) limits vaccine efficacy^26^ and may reflect an increased risk for infectious disease morbidity and mortality^27^. The main finding of the present study was that %BF had a significant effect on the antibody response after accounting for the effects of covariates that could impact this response including age, time since vaccination, and the type of vaccine received (Fig. 3). Our statistical models demonstrated that %BF was an independent predictor of S1 and RBD antibodies present in the serum following SARS-CoV-2 mRNA vaccination. Specifically, in people with higher %BF there was a significantly lower amount of both the RBD and S1 antibodies present in the serum. While we lack sufficient evidence to suggest that an individual’s %BF has a statistically significant effect on the amount of S2 antibody present in the serum (*p* = 0.106), we demonstrated that there was still a negative effect (*β* =  − 190.08) of %BF on the S2 antibody response. Therefore, the effect of %BF on the S2 antibody response may indeed be of practical significance and the directionality of the relationship between %BF and S2 antibody response is in agreement with the responses observed for the S1 and RBD antibodies. The current study lends support that as %BF increases there is a decreased efficacy of SARS-CoV-2 mRNA vaccination, possibly leading to a greater risk of an individual contracting SARS-CoV-2 and developing COVID-19 regardless of their age, type of mRNA vaccine received, and days since vaccination. Therefore, although vaccination is one of our primary strategies against viruses such as SARS-CoV-2, this data suggests that higher %BF could reduce the level of circulating SARS-CoV-2-specific IgG antibodies following SARS-CoV-2 mRNA vaccination.

Obesity is characterized by chronic low-grade inflammation^28^ and is a risk factor for increased morbidity and mortality amongst SARS-CoV-2 infected patients^29,30^. In one study^31^, obese high fat-fed mice developed elevated markers of inflammation that were associated with lower levels of influenza-specific neutralizing antibodies as well as defective generation of effector memory CD8 + T cells after vaccination. The authors discussed the possibility that the impaired memory T-cell response could have influenced the poorer maintenance of neutralizing antibodies observed in their study^31^. There is also evidence that obesity interferes with B-cell responses furthering the autoimmune inflammatory response following SARS-CoV-2 infection and increasing the severity of COVID-19^32^. Aside from the mentioned physiological/immunological factors that could contribute to reduced vaccine effectiveness, anatomical factors in people with higher body fatness may also reduce effectiveness. For example, as individuals increase in weight and %BF, longer needle lengths may be required in order to ensure that the vaccine is deposited intramuscularly^33^. Although vaccination amongst obese individuals will likely still result in some vaccine-conferred protection, needles that are too short and not individualized to the individual’s level of body fatness may result in the vaccine being deposited into a fat pad, which could reduce the immunogenic response to the vaccine^33^. In agreement with these observations, previous researchers have demonstrated that excess body fat and obesity are associated with an impaired immune response and poor efficacy of other vaccines such as those against the hepatitis B virus^34,35^, hepatitis A virus^36,37^, and the influenza A virus^10–12^. Importantly, these previous vaccines elicited an immune response via inactive, attenuated, or recombinant viral proteins and are different than the two vaccines evaluated in the current study (Moderna and Pfizer) which are both lipid-encapsulated mRNA vaccines. Therefore, the current study builds upon previous findings by extending them to SARS-CoV-2 mRNA vaccines (Moderna and Pfizer) and demonstrates that the effect of an individual’s %BF on the magnitude of their antibody response is independent of known factors that influence vaccine-induced immunity such as the individual’s age and time since vaccination^5^. Importantly, chronic low level inflammation associated with obesity impairs the generation of antibodies following influenza vaccination^31^. This may explain the inverse relationship between %BF and antibody responses observed in the current study following SARS-CoV-2 mRNA vaccination. Given the obesity epidemic and the fact that obesity is an independent risk factor for severe COVID-19, there is a need to enact strategies for increasing the efficacy of SARS-CoV-2 vaccines amongst this population. Based on the present results, one effective lifestyle-related strategy may be to reduce excess body fat. Although obesity can have a biological component and is therefore not always modifiable, there are behavioral and environmental factors that contribute to the development of obesity that can be modified. Proper nutritional and exercise interventions are often recommended as effective strategies for combating and preventing obesity. Decreasing excess body fat can improve the likelihood that the vaccine is deposited intramuscularly that may increase its immunogenic effect^33^. Evidence supports that decreasing excess body fat can also decrease proinflammatory markers^28^, therefore potentially protecting the generation of effector memory T-cells and neutralizing antibodies following vaccination^31,32^. Results of the current study might indicate that decreasing excess body fat can positively affect the immunogenic effect of vaccination, a notion supported by recent evidence^14^.

Participating in regular exercise and being physical active is recommended for all adults^38^. The negative health consequences of failing to meet adequate levels of physical activity are myriad and include a greater risk of COVID-19 leading to hospitalization and death^39^. Whether physical activity levels impact the efficacy of prophylactic immune response to SARS-CoV-2 vaccination is not clear, but there is growing evidence that regular physical activity increases the efficacy of vaccination^16^. While we did not observe a statistically significant effect of physical activity on the magnitude of S1 (*p* = 0.127), RBD (*p* = 0.081), and S2 (*p* = 0.120) antibodies following SARS-CoV-2 mRNA vaccination, in our a priori power analysis we assumed a moderate effect size (*f*^*2*^ = 0.15) between our predictor and outcome variables which may have overestimated the relationship between physical activity and antibody responses resulting in the current study being slightly underpowered to identify a statistically significant effect. Though not statistically significant, there was a positive effect of the average time spent being physical active on the magnitude of antibody response for S1 (*β* = 0.27), RBD (*β* = 0.19), and S2 (*β* = 0.23) suggesting that physical activity may be of practical significance for improving an individual’s antibody response following vaccination. In support of this, previously sedentary aged adults (~ 70 years) who were randomly assigned to a sham intervention had poorer influenza vaccine responses, while those assigned to a cardiovascular exercise program had a significantly greater seroprotection rate at 24 weeks after vaccination^40^. Also, in a recent preprint, individuals who were more physically active had a greater S1/S2 antibody response following vaccination with an inactivated SARS-CoV-2 virus vaccine (CoronaVac)^41^. The current study builds upon these findings by suggesting that increasing an individual’s weekly physical activity may potentially improve the antibody response following mRNA vaccinations as well.

## Conclusions

With the increasing number of SARS-CoV-2 infections and variants, along with the known waning effects of vaccination, future mRNA vaccinations such as boosters are encouraged to sustain immunity. Our findings demonstrate that as %BF increases there is a decreased efficacy of SARS-CoV-2 mRNA vaccination, characterized as decreased S1, S2, and RBD antibodies present in the serum, which can lead to a greater risk of contracting SARS-CoV-2 and developing COVID-19 regardless of the individual’s age, type of mRNA vaccine received, and time since vaccination. Therefore, decreasing excess body fat may be an effective strategy to improve the prophylactic immune response following mRNA vaccination. In addition, increased physical activity may be a beneficial strategy to improve the antibody response following mRNA vaccination, but more research is warranted with larger sample sizes to substantiate this effect. These findings highlight several behavioral changes (reducing excess body fat and increasing physical activity) that individuals can take that may improve the efficacy of SARS-CoV-2 mRNA vaccination.

## Data Availability

The data used to support the findings of this study are available from the corresponding author upon request.
